# Body mass index and gastric cancer risk: results from the Stomach Cancer Pooling Project Consortium

**DOI:** 10.1093/ije/dyaf160

**Published:** 2025-09-28

**Authors:** Roberta Pastorino, Denise Pires Marafon, Angelica Valz Gris, Nicolò Lentini, Antonio Cristiano, Nuria Aragonés, Vicente Martín, David Zaridze, Dmistry Maximovich, Jesus Vioque, Sandra Gonzalez-Palacios, Reza Malekzadeh, Farhad Pourfarzi, Joshua Muscat, Mary H Ward, Charles S Rabkin, Eva Negri, Rossella Bonzi, Carlo Pelucchi, Paolo Boffetta, Maria Costanza Camargo, Maria Paola Curado, Nuno Lunet, Zuo-Feng Zhang, Carlo La Vecchia, Stefania Boccia

**Affiliations:** Section of Hygiene, University Department of Health Sciences and Public Health, Università Cattolica del Sacro Cuore, Rome, Italy; Department of Woman and Child Health and Public Health, Fondazione Policlinico Universitario A. Gemelli IRCCS, Rome, Italy; Section of Hygiene, University Department of Health Sciences and Public Health, Università Cattolica del Sacro Cuore, Rome, Italy; Section of Hygiene, University Department of Health Sciences and Public Health, Università Cattolica del Sacro Cuore, Rome, Italy; Section of Hygiene, University Department of Health Sciences and Public Health, Università Cattolica del Sacro Cuore, Rome, Italy; Section of Hygiene, University Department of Health Sciences and Public Health, Università Cattolica del Sacro Cuore, Rome, Italy; Cancer Epidemiology Section, Public Health Division, Department of Health of Madrid, Madrid, Spain; Consortium for Biomedical Research in Epidemiology and Public Health (CIBERESP), Madrid, Spain; Consortium for Biomedical Research in Epidemiology and Public Health (CIBERESP), Madrid, Spain; Research Group in Gene-Environment Interactions and Health, University of León, León, Spain; Department of Clinical Epidemiology, N.N.Blokhin National Medical Research Center for Oncology, Moscow, Russia; Department of Clinical Epidemiology, N.N.Blokhin National Medical Research Center for Oncology, Moscow, Russia; Consortium for Biomedical Research in Epidemiology and Public Health (CIBERESP), Madrid, Spain; Instituto de Investigación Sanitaria y Biomédica de Alicante, Universidad Miguel Hernandez (ISABIAL-UMH), Alicante, Spain; Consortium for Biomedical Research in Epidemiology and Public Health (CIBERESP), Madrid, Spain; Instituto de Investigación Sanitaria y Biomédica de Alicante, Universidad Miguel Hernandez (ISABIAL-UMH), Alicante, Spain; Digestive Oncology Research Center, Digestive Disease Research Institute, Tehran University of Medical Sciences, Tehran, Iran; Digestive Disease Research Center, Ardabil University of Medical Sciences, Ardabil, Iran; Department of Public Health Sciences, Pennsylvania State University College of Medicine, Hershey, PA, United States; Division of Cancer Epidemiology and Genetics, National Cancer Institute, Rockville, MD, United States; Division of Cancer Epidemiology and Genetics, National Cancer Institute, Rockville, MD, United States; Department of Medical and Surgical Sciences, University of Bologna, Bologna, Italy; Department of Clinical Sciences and Community Health, University of Milan, Milan, Italy; Department of Clinical Sciences and Community Health, University of Milan, Milan, Italy; Department of Medical and Surgical Sciences, University of Bologna, Bologna, Italy; Stony Brook Cancer Center, Stony Brook University, Stony Brook, NY, United States; Division of Cancer Epidemiology and Genetics, National Cancer Institute, Rockville, MD, United States; Centro Internacional de Pesquisa, A. C. Camargo Cancer Center, São Paulo, Brazil; EPIUnit—Instituto de Saúde Pública da Universidade do Porto, Porto, Portugal; Laboratório para a Investigação Integrativa e Translacional em Saúde Populacional (ITR), Porto, Portugal; Departamento de Ciências da Saúde Pública e Forenses e Educação Médica, Faculdade de Medicina da Universidade do Porto, Porto, Portugal; Department of Epidemiology, UCLA Fielding School of Public Health and Jonsson Comprehensive Cancer Center, Los Angeles, CA, United States; Department of Clinical Sciences and Community Health, University of Milan, Milan, Italy; Section of Hygiene, University Department of Health Sciences and Public Health, Università Cattolica del Sacro Cuore, Rome, Italy; Department of Woman and Child Health and Public Health, Fondazione Policlinico Universitario A. Gemelli IRCCS, Rome, Italy

**Keywords:** gastric cancer, body mass index, cancer prevention

## Abstract

**Background:**

Body mass index (BMI) has been associated with gastric cancer (GC), though results are conflicting regarding the GC subsites of cardia and non-cardia. This study aims to evaluate the associations between BMI and GC risk, focusing on these distinct anatomical subsites.

**Methods:**

We pooled data from seven case–control studies from the Stomach Cancer Pooling (StoP) Project. Pooled odds ratios (ORs) and the corresponding 95% confidence intervals (CIs) of GC risk across BMI categories (normal weight, overweight, obesity) were calculated by pooling study-specific ORs through random-effects meta-analytic models. The dose–response relationship between BMI and the risk of GC cancer was assessed by using a one-stage mixed-effects logistic regression model. Results were stratified according to cardia and non-cardia GC.

**Results:**

The analysis comprised 1478 GC cases, including 511 cardia and 967 non-cardia cases, and 6671 controls. There was an increased risk of cardia GC among obese patients (OR 1.57, 95% CI 1.20–2.06), while no association was found for non-cardia GC (OR 0.82, 95% CI 0.66–1.01). Restricting the analysis to population-based studies, the association for cardia GC became stronger for obese (OR 1.65, 95% CI 1.09–2.48) and overweight (OR 1.62, 95% CI 1.10–2.39) patients. The dose–response meta-analysis showed an increased risk of cardia GC with increasing BMI values, ranging from a null effect at a BMI of 21.75 to an OR of 2.06 (95% CI 1.22–3.48) for a BMI of ≥40.

**Conclusion:**

Our results indicate an association between higher BMI categories and the risk of cardia GC, whereas no association was found with non-cardia GC.

Key Messages

- This pooled analysis of seven case–control studies investigated the association between body mass index (BMI) and the risk of gastric cancer (GC), with a focus on cardia and non-cardia subsites.
- An increased risk of cardia GC was observed among overweight and obese individuals, whereas no association was found for non-cardia GC.
- These findings support the hypothesis that excess body weight contributes to the development of cardia GC and highlight the importance of tailored prevention strategies.

## Background

Gastric cancer (GC) remains a major global health concern, affecting over 1 million people and causing an estimated 660 000 deaths in 2022. It ranks fifth in incidence and fourth in mortality globally [1]. Despite the declining incidence and mortality rates in most high-income countries over recent decades, GC continues to pose a substantial health challenge worldwide.

The carcinogenesis of GC is influenced by both genetic and environmental factors [2]. These risk factors include *Helicobacter pylori* infection, low socioeconomic status, dietary habits, alcohol consumption, tobacco smoking, gastroesophageal reflux disease, family history of gastrointestinal malignancies, and inherited predisposition [3–5].

Histologically, most GCs are adenocarcinomas, with two major histologic types: diffuse and intestinal [5]. Topographically, GC is mainly classified into cardia and non-cardia stomach cancer, which exhibit different etiological and epidemiological patterns [6].

In recent decades, the global prevalence of overweight and obesity has escalated, posing a major public health concern [7]. This increase in obesity rates is associated with various lifestyle changes, including decreased physical activity and increased consumption of energy-dense foods. The rise in obesity is a critical issue due to its association with several clinical conditions and noncommunicable diseases, including cancer. Excess body fat is thought to affect cancer risk through mechanisms such as insulin resistance, inflammation, and alterations in the levels of growth factors and sex hormones [8, 9].

Current evidence suggests a potential association between GC, particularly the cardia subsite, and high body weight, as defined by the body mass index (BMI) [10–15]. However, conflicting results between different meta-analyses have been noteworthy, especially when cardia cancers and non-cardia cancers have been analysed together [10–13, 16, 17].

This study aims at evaluating the associations between BMI and GC risk, with a focus on the two distinct subsites of cardia and non-cardia GC, by conducting a pooled analysis within the Stomach Cancer Pooling (StoP) Project [18, 19].

## Methods

### Study population

This study is an individual participant data meta-analysis based on data collected from studies conducted within the StoP Project. The methodology used by the StoP Consortium has been previously published and recently updated [19, 20]; further details are available in the Supplementary Material.

For the current analysis, a total of 8149 subjects (1478 cases and 6671 controls) enrolled in seven study centers from five countries (Iran, Italy, Russia, Spain, and the USA) were included [20–26] (Supplementary Table 1). These studies were selected based on the availability of necessary data on height and weight. For cases, this information had to date back to ≥1 year before diagnosis, while studies collecting data on weight at diagnosis of GC were excluded to limit the potential role of reverse causation [27]. Participants with cancer overlapping or for whom the location was not specified were excluded from the analysis.

### Exposure definition

The BMI of the population under study, defined as the body weight divided by the square of the body height (kg/m^2^), was calculated by using data on weight and height as reported in the structured questionnaires by the participants. Depending on the specific studies and the information requested in the questionnaire, the weight for cases was reported as either the weight from ≥1 year prior to diagnosis [22–26] or the maximum weight recorded during their lifetime [20, 21] (Supplementary Table 1). For controls, the weight was reported at hospital admission (for controls in hospital-based case–control studies) or recruitment (for controls in population-based case–control studies).

Study participants were categorized into BMI categories according to the World Health Organization (WHO) categories [28], with individuals having a BMI of between 25 and 29.9 identified as ‘overweight’, those with a BMI of ≥30 designated as ‘obesity’, individuals with a BMI ranging from 18.5 to 24.9 classified as ‘normal weight’, and those with a BMI of <18.5 considered ‘underweight’.

### Statistical analysis

A two-stage model was performed to estimate the association between BMI and GC, separately for cardia and non-cardia GC. In the first stage, for each study, we estimated the odds ratios (ORs) and the corresponding 95% confidence intervals (CIs) of GC by comparing individuals across different BMI categories to those with a normal BMI, through multivariable logistic regression models. The models included terms for sex, age at diagnosis/interview as a continuous variable, smoking status (never, former, current smoker), socioeconomic status (study-specific low, intermediate, high as defined in each original study based on education, income, or occupation), and alcohol drinking (never, low ≤12 g/day, intermediate >12 and ≤47 g/day, high >47 g/day). These adjustment variables were selected based on their significance in univariate analysis (*P* < .15) and a missing-data percentage of <10%. For covariates with ≤10% missing values (smoking status, socioeconomic status, and alcohol-drinking status), we performed multiple imputations by using full chained equations, generating 10 imputed datasets for each study. Each imputation model included the same set of covariates and outcome as the analysis model, and imputation results were combined by using Rubin’s rule [29]. In the second stage, summary (pooled) effect estimates were computed by using random-effects models. Heterogeneity between studies was quantified by using *I*^2^ (%) statistics [30].

In addition, a one-stage analysis with intercept as the random variable was performed to estimate the association between BMI and GC with a generalized linear mixed model with logistic link function estimated by restricted maximum likelihood with nine quadrature points for adaptive Gauss–Hermite approximation.

Stratified analyses were conducted to evaluate whether the effect of the BMI categories on GC varied across subgroups, defined by age at diagnosis, sex, education, socioeconomic status, smoking status, alcohol-drinking status, fruit-and-vegetables intake categorized by study-specific tertiles [31], salt intake categorized by study-specific tertiles [32], family history, histological type, *H. pylori* infection status determined by serologic test, history of diabetes, and type of control. For each stratifying variable, Q statistics were computed to test the heterogeneity across the strata [33].

We computed the relative excess risk due to interaction (RERI) from a one-stage logistic model to assess the occurrence of additive interaction between the exposure and the smoking and alcohol-drinking status in relation to the risk of cardia GC, variables for which heterogeneity across the strata was detected. Results were presented by following the methods of Knol and Vander Weele [34].

We modeled the dose–response relationship between BMI prior to diagnosis and the risk of cardia cancer by using a one-stage mixed-effects logistic regression model that included the same set of covariates as previously described and a random intercept for each study. To account for potential nonlinearity, we incorporated fractional polynomials to flexibly characterize the relationship between BMI and cardia cancer risk, selecting the optimal model based on the lowest deviance. Additionally, the ORs were specifically chosen for values of BMI based on both biological plausibility and statistical considerations.

Focusing on obese individuals, we conducted an additional analysis, dividing them into Obesity Class I (BMI = 30.0–34.9) and Obesity Class II/III (BMI ≥35.0), to investigate the association between BMI and GC in these subcategories.

We conducted sensitivity analyses that were restricted to studies with population-based controls to assess the potential impact of Berkson bias due to the inclusion of hospital-based individuals [28]. We also performed a sensitivity analysis by using a two-stage model with multivariable logistic regressions, additionally adjusting for fruit-and-vegetables intake and salt intake, to assess potential residual confounding due to these dietary factors. One study (16-USA) was excluded because data on these variables were not available.

Statistical analyses were carried out by using Stata software version 17 (StataCorp LLC) and RStudio version 4.4.3.

## Results

Table 1 presents the main characteristics of 1478 GC cases (511 cardia GC and 967 non-cardia GC) and 6671 controls (2579 hospital- and 4092 population-based) included in the analysis, while detailed information of each study is reported in Supplementary Table 2a and b.

**Table 1.** Main characteristics of cardia and non-cardia GC cases and controls in the StoP Project Consortium studies included in the analysis.

	Control (*N* = 6671)		Cardia (*N* = 511)		Non-cardia (*N* = 967)		Total (*N* = 8149)	
	*n*	%	*n*	%	*n*	%	*n*	%
Study								
03-Italy	523	7.8	10	2.0	91	9.4	624	7.7
09-Russia	504	7.6	78	15.3	165	17.1	747	9.2
10-Iran	390	5.8	114	22.3	81	8.4	585	7.2
16-USA	1322	19.8	158	30.9	2	0.2	1482	18.2
21-Spain	3056	45.8	91	17.8	311	32.2	3458	42.4
23-Spain	397	6.0	28	5.5	213	22.0	638	7.8
32-USA	479	7.2	32	6.3	104	10.8	615	7.5
Sex								
Male	4107	61.6	398	77.9	581	60.1	5086	62.4
Female	2564	38.4	113	22.1	386	39.9	3063	37.6
Age (years)								
<65	3493	52.4	250	48.9	396	41.0	4139	50.8
≥65	3178	47.6	261	51.1	571	59.0	4010	49.2
BMI								
Underweight	81	1.2	2	0.4	11	1.1	94	1.2
Normal weight	2417	36.2	155	30.3	337	34.9	2909	35.7
Overweight	2827	42.4	231	45.2	437	45.2	3495	42.9
Obesity	1346	20.2	123	24.1	182	18.8	1651	20.3
Socioeconomic status								
Low	2560	38.4	204	39.9	531	54.9	3295	40.4
Intermediate	2696	40.4	203	39.7	331	34.2	3230	39.6
High	1376	20.6	104	20.4	103	10.7	1583	19.4
Missing	39	0.6	0	0.0	2	0.2	41	0.5
Smoking status								
Never	3042	45.6	190	37.2	482	49.8	3714	45.6
Former	2222	33.3	172	33.7	239	24.7	2633	32.3
Current	1383	20.7	146	28.6	244	25.2	1773	21.8
Missing	24	0.4	3	0.6	2	0.2	29	0.4
Alcohol-drinking status								
Never	1772	26.6	173	33.9	300	31.0	2245	27.5
Low	1883	28.2	84	16.4	180	18.6	2147	26.3
Intermediate	1524	22.8	89	17.4	225	23.3	1838	22.6
High	962	14.4	140	27.4	174	18.0	1276	15.7
Missing	530	7.9	25	4.9	88	9.1	643	7.9
Fruit-and-vegetables intake								
Low	1398	21.0	53	10.4	225	23.3	1676	20.6
Intermediate	1625	24.4	117	22.9	311	32.2	2053	25.2
High	1886	28.3	162	31.7	347	35.9	2395	29.4
Missing	1762	26.4	179	35.0	84	8.7	2025	24.8
History of diabetes								
No	5497	82.4	322	63.0	685	70.8	6504	79.8
Yes	761	11.4	41	8.0	91	9.4	893	11.0
Missing	413	6.2	148	29.0	191	19.8	752	9.2
*Helicobacter pylori* infection status								
Negative	522	7.8	70	13.7	84	8.7	676	8.3
Positive	2098	31.4	156	30.5	311	32.2	2565	31.5
Missing	4051	60.7	285	55.8	572	59.2	4908	60.2
Family history of GC								
No	4949	74.2	311	60.9	797	82.4	6057	74.3
Yes	351	5.3	36	7.0	146	15.1	533	6.5
Missing	1371	20.6	164	32.1	24	2.5	1559	19.1
Histological type								
Intestinal			161	31.5	407	42.1		
Diffuse			69	13.5	287	29.7		
Mixed/undifferentiated			229	44.8	164	17.0		
Missing			52	10.2	109	11.3		
Type of control								
Population-based	4092	61.3						
Hospital-based	2579	38.7						

BMI: Body Max Index. GC: Gastric Cancer. USA: United State of America.

The proportions of overweight and obese cases were 45.2% and 24.1%, respectively, for cardia GC and 45.2% and 18.8% for non-cardia GC, while, in controls, these percentages were 42.4% and 20.2%. Compared with controls, cases had higher proportions of current smokers (20.7% controls vs 28.6% cardia GC and 25.2% non-cardia GC). Cardia GC cases had a higher proportion of males (77.9% vs 61.6% controls and 60.1% non-cardia GC), whereas non-cardia GC cases had a higher proportion of patients with a family history of GC (15.1% vs 5.3% controls and 7.0% cardia GC).

**Figure 1.**
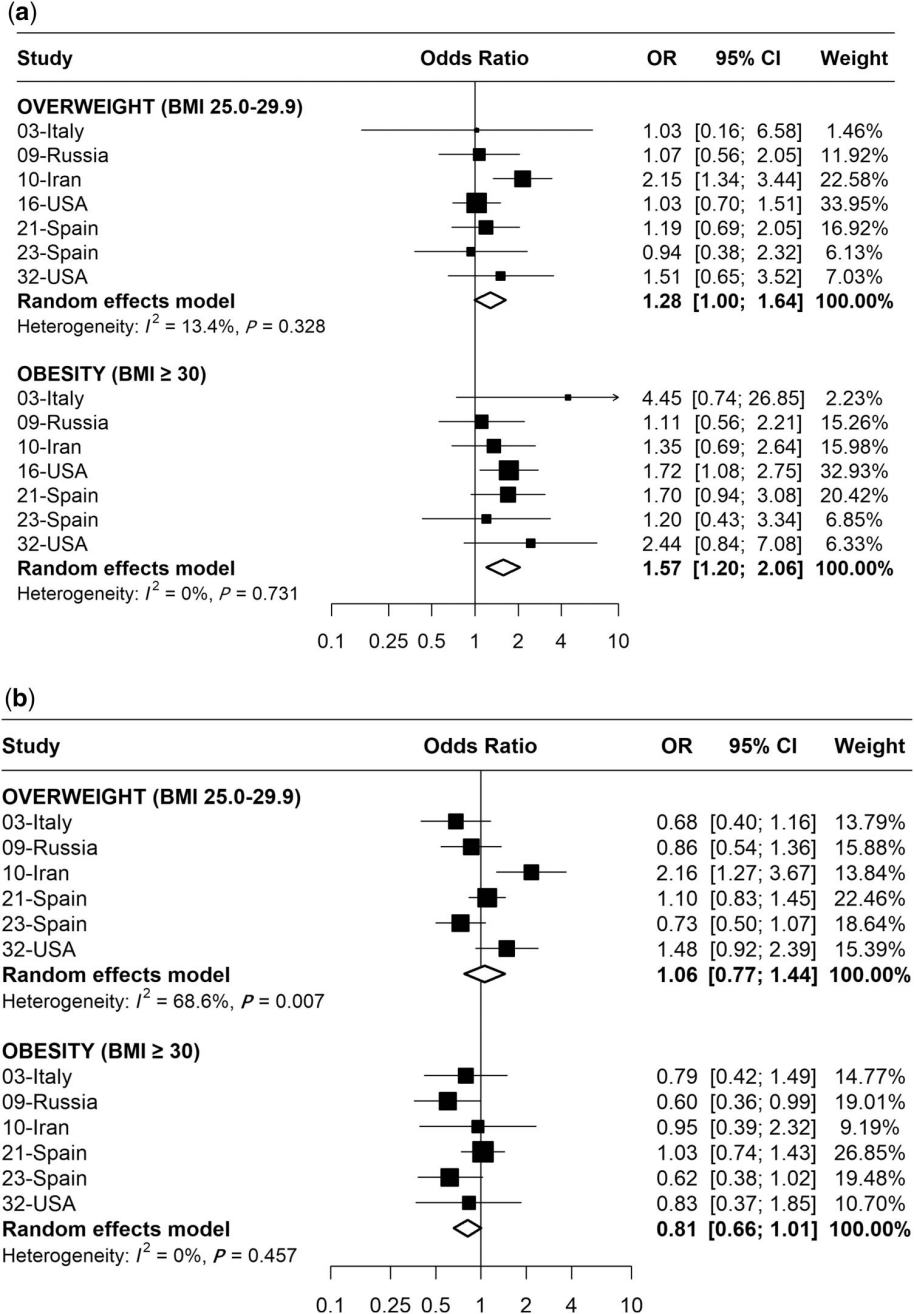
Study-specific and adjusted pooled ORs and corresponding 95% CIs of (a) cardia GC risk and (b) non-cardia GC risk for overweight and obese individuals compared with controls, respectively. The number of individuals categorized as underweight (*N* = 94) is insufficient for the meta-analysis.

Figure 1 shows the pooled overall ORs and the corresponding 95% CIs for the association between BMI and GC, separately for cardia and non-cardia GC. The pooled ORs for overweight individuals compared with those with normal weight were 1.28 (95% CI 1.00–1.64) for cardia GC and 1.06 (95% CI 0.77–1.44) for non-cardia GC. Obese individuals showed an increased risk of cardia GC (OR 1.57, 95% CI 1.20–2.06), whereas no association was found for non-cardia GC (OR 0.81, 95% CI 0.66–1.01). Due to the limited number of underweight individuals in the dataset (*N* = 94), their association with the risk of GC could not be evaluated.

Substantial heterogeneity was observed among the studies in the analysis of non-cardia GC for overweight individuals (*I*^2^=68.6%, *P *= .007). The Galbraith plot (Supplementary Figure 1) identified the studies conducted in Italy, Iran, and Spain [22, 23, 26] as potential sources of heterogeneity. After the exclusion of these studies, the heterogeneity decreased (*I*^2^=22.4%, *P *= .276) without an appreciable change in the results (OR 1.11, 95% CI 0.86–1.43). The pattern of findings was similar for the analysis without imputed data (data not shown).

The one-stage approach consistently found that overweight and obese individuals have an increased risk of cardia GC, whereas we did not observe associations for non-cardia GC (Supplementary Figure 2).

The stratified analysis is presented in detail in Table 2. Regarding non-cardia cases, the analysis showed a similar effect of BMI among the strata of all the variables analysed. Regarding the risk of cardia cancer, an increased odds of GC for overweight individuals was observed for non-smokers (OR 1.92, 95% CI 1.29–2.85) (Q = 69.2%, *P *= .039). Similarly, an increased odds of GC for overweight individuals was observed among nondrinkers (OR 2.20, 95% CI 1.48–3.27) (Q = 80.8%, *P *= .001). An increased risk of cardia GC associated separately with smoking and alcohol-drinking status and overweight/obesity was confirmed in the RERI analysis (Tables 3 and 4**)**. However, no positive or negative additive interaction was identified for smoking and alcohol-drinking status and overweight or obesity (Tables 3 and 4).

**Table 2.** Pooled adjusted^a^ ORs with 95% CIs for the association between GC and overweight and obesity BMI categories in the StoP Project, overall and stratified by participants’ characteristics.

	Overweight						Obesity					
	Cardia			Non-cardia			Cardia			Non-cardia		
	Number of studies (cases/controls)	Adjusted OR (95% CI)	*I* ^2^ (%)	Number of studies (cases/controls)	Adjusted OR (95% CI)	*I* ^2^ (%)	Numb er of studies (cases/controls)	Adjusted OR (95% CI)	*I* ^2^ (%)	Number of studies (cases/controls)	Adjusted OR (95% CI)	*I* ^2^ (%)
Overall	7 (386/5244)	1.28 (1.00–1.64)	13.4	6 (774/5244)	1.06 (0.77–1.44)	68.6	7 (278/3763)	1.57 (1.20–2.06)	0.0	6 (519/3070)	0.81 (0.67–1.01)	0.0
Age at diagnosis (years)												
<65	6 (173/2489)	1.21 (0.86–1.70)	0.0	6 (302/2060)	0.82 (0.51–1.31)	63.6	6 (138/1911)	1.88 (1.16–3.03)	24.5	6 (246/1585)	0.83 (0.60–1.13)	0.0
≥65	7 (211/2529)	1.27 (0.91–1.78)	11.8	6 (470/2103)	1.38 (1.06–1.80)	19.1	6 (130/1610)	1.23 (0.80–1.90)	0.0	6 (273/1485)	0.88 (0.65–1.17)	0.0
Sex												
Male	7 (301/3270)	1.14 (0.88–1.47)	0.0	6 (483/2377)	1.09 (0.75–1.59)	64.2	7 (219/2108)	1.67 (1.23–2.27)	1.1	6 (290/1568)	0.76 (0.54–1.06)	23.6
Female	6 (84/1783)	1.95 (1.18–3.24)	0.0	6 (290/1786)	0.94 (0.66–1.34)	33.5	5 (54/1434)	1.44 (0.73–2.83)	0.0	5 (222/1424)	0.80 (0.50–1.27)	41.8
Socioeconomic status												
Low	6 (158/1917)	0.94 (0.46–1.94)	60.0	6 (424/1823)	1.15 (0.66–2.01)	71.4	5 (101/1278)	1.28 (0.82–2.01)	0.0	5 (270/1334)	0.82 (0.62–1.09)	0.0
Intermediate	7 (141/2141)	1.22 (0.84–1.79)	0.0	6 (259/1480)	0.82 (0.56–1.21)	31.6	5 (110/1470)	2.20 (1.14–4.26)	44.2	6 (195/1104)	0.85 (0.59–1.23)	2.9
High	5 (84/1054)	0.98 (0.60–1.60)	0.0	5 (86/853)	1.18 (0.68–2.06)	9.0	4 (57/733)	1.52 (0.79–2.91)	0.0	4 (46/605)	0.59 (0.25–1.48)	0.0
Smoking status		^b^										
Never	7 (137/2357)	1.92 (1.29–2.85)	0.0	6 (378/1985)	1.00 (0.78–1.28)	0.0	6 (93/1623)	1.93 (1.16–3.22)	0.0	6 (255/1511)	0.81 (0.60–1.09)	0.0
Former	6 (133/1618)	1.05 (0.70–1.58)	3.5	6 (191/1264)	1.15 (0.80–1.66)	0.0	6 (92/1130)	1.25 (0.76–2.04)	0.0	6 (117/929)	0.88 (0.49–1.60)	32.2
Current	5 (110/996)	0.97 (0.63–1.50)	0.0	6 (203/914)	0.80 (0.45–1.42)	57.9	6 (88/747)	1.55 (0.88–2.72)	26.5	6 (147/630)	0.81 (0.44–1.48)	33.0
Drinking status		^c^										
Never	6 (138/1269)	2.20 (1.48–3.27)	0.0	6 (238/1110)	1.33 (0.81–2.17)	55.6	5 (80/888)	1.86 (0.90–3.85)	23.9	6 (161/890)	0.86 (0.48–1.55)	45.0
Low	3 (57/1356)	0.86 (0.49–1.51)	0.0	5 (144/1232)	0.80 (0.55–1.18)	0.0	4 (46/1031)	1.72 (0.83–3.55)	12.0	4 (98/887)	0.81 (0.50–1.31)	0.0
Intermediate	5 (62/1087)	1.49 (0.83–2.65)	0.0	5 (177/1029)	0.80 (0.44–1.47)	62.8	5 (45/718)	2.12 (1.06–4.24)	0.8	5 (127/687)	0.69 (0.44–1.08)	0.0
High	6 (105/742)	0.72 (0.46–1.13)	0.0	5 (144/449)	0.94 (0.61–1.44)	0.0	5 (85/470)	1.10 (0.66–1.85)	0.0	5 (83/327)	0.50 (0.28–0.87)	0.0
Fruit-and-vegetables intake												
Low	6 (37/1084)	1.62 (0.73–3.60)	0.0	6 (187/1084)	0.74 (0.52–1.06)	0.0	4 (25/684)	2.95 (0.65–13.49)	53.1	4 (126/755)	0.69 0.38–1.25)	27.3
Intermediate	5 (95/1164)	1.33 (0.72–2.48)	30.6	6 (251/1294)	1.16 (0.71–1.88)	56.4	4 (52/790)	0.92 (0.48–1.78)	0.0	6 (155/882)	0.80 (0.45–1.43)	47.9
High	5 (116/1316)	1.21 (0.67–2.20)	38.1	6 (275/1462)	1.18 (0.72–1.94)	64.2	6 (95/1126)	1.42 (0.87–2.33)	0.0	6 (189/1126)	0.81 (0.52–1.27)	27.1
Salt intake												
Low	6 (95/1661)	1.21 (0.78–1.90)	0.0	6 (284/1661)	1.01 (0.77–1.33)	0.0	6 (65/1220)	0.98 (0.54–1.79)	0.0	6 (191/1220)	0.73 (0.51–1.04)	0.0
Intermediate	5 (97/1222)	1.39 (0.83–2.32)	0.0	6 (269/1302)	0.96 (0.70–1.30)	0.0	5 (73/891)	2.15 (1.12–4.10)	9.2	6 (184/948)	0.83 (0.55–1.26)	9.7
High	3 (51/772)	1.42 (0.70–2.86)	0.0	4 (133/807)	0.85 (0.57–1.27)	0.0	3 (35/562)	1.59 (0.68–3.71)	0.0	3 (89/570)	0.74 (0.44–1.26)	0.0
GC family history												
No	6 (233/3868)	1.48 (1.09–2.02)	5.9	6 (636/3868)	1.01 (0.72–1.42)	68.4	6 (166/2846)	1.64 (1.12–2.41)	13.4	6 (435/2846)	0.81 (0.64–1.02)	0.0
Yes	3 (26/207)	0.82(0.29–2.28)	23.5	6 (118/260)	0.96 (0.55–1.66)	0.0	2 (13/130)	0.40 (0.07–2.26)	0.0	5 (63/178)	0.77(0.39–1.52)	0.0
GC histological type												
Intestinal	6 (121/4163)	1.50 (0.93–2.41)	22.2	6 (332/4163)	1.40 (0.86–2.87)	38.7	6 (87/3070)	1.68 (1.05–2.68)	0.0	6 (208/3070)	0.91(0.59–1.41)	32.3
Diffuse	4 (48/3461)	1.24 (0.67–2.33)	0.0	6 (237/4163)	0.89 (0.65–1.23)	20.4	5 (40/2787)	1.33 (0.65–2.72)	0.0	6 (163/3070)	0.67 (0.46–0.98)	0.0
Mixed/undifferentiated	6 (178/4163)	1.31 (0.77–2.22)	30.3	6 (125/4163)	0.98 (0.66–1.46)	0.0	5 (120/1816)	1.55 (1.31–2.32)	0.0	4 (81/1532)	0.63 (0.38–1.03)	0.0
*Helicobacter pylori* infection status												
Negative	3 (49/400)	1.32 (0.37–4.65)	64.4	3 (59/400)	1.27 (0.26–6.27)	65.6	3 (39/322)	1.42 (0.56–3.58)	22.4	3 (44/322)	1.58 (0.34–7.35)	0.0
Positive	3 (125/1641)	1.53 (0.91–2.56)	28.2	3 (248/1641)	1.22 (0.65–2.28)	72.1	3 (76/1159)	1.15 (0.68–1.95)	0.0	3 (154/1159)	0.78 (0.54–1.12)	0.0
History of diabetes												
No	5 (238/4044)	1.04 (0.79–1.38)	0.0	4 (542/3055)	0.89 (0.71–1.11)	15.1	5 (183/2838)	1.54 (1.10–2.16)	0.0	4 (379/2216)	0.79 (0.57–1.11)	42.4
Yes	3 (22/410)	1.11 (0.28–4.36)	40.9	3 (57/333)	0.94 (0.48–1.84)	0.0	2 (18/317)	1.73 (0.54–5.58)	0.0	3 (48/290)	0.56 (0.18–1.75)	31.1
Type of control												
Population-based	3 (187/3129)	1.62 (1.10–2.39)	24.5	3 (406/3129)	1.44 (0.98–2.14)	61.6	3 (120/2269)	1.65 (1.09–2.48)	0.0	3 (252/2269)	0.99 (0.75–1.32)	0.0
Hospital-based	4 (199/2115)	1.03 (0.76–1.40)	0.0	4 (366/1034)	0.76 (0.58–0.98)	0.0	4 (158/1494)	1.52 (1.07–2.17)	0.0	4 (267/801)	0.65 (0.48–0.88)	0.0

a ORs are adjusted for age, sex, study, smoking status, alcohol-drinking status, and socioeconomic status.

b Cochrane Q statistics for heterogeneity across strata: Q = 69.2%, *P *= .039.

c Cochrane Q statistics for heterogeneity across strata: Q = 80.8%, *P *= .001.

**Table 3.** Interaction analysis between BMI (overweight or obesity vs normal weight) and smoking status (current or former vs never smoker) in cardia GC.

	Cardia GC (*N* = 251/3514)			Cardia GC (*N* = 185/2545)		
	Normal weight	Overweight	RERI	Normal weight	Obesity	RERI
	OR (95% CI)	OR (95% CI)	(95% CI)	OR (95% CI)	OR (95% CI)	(95% CI)
Smoking status						
Never	1.00	1.92 (1.30, 2.83)	–0.86 (–2.10; 0.38)	1.00	2.17 (1.40; 3.37)	0.44 (–1.31; 2.20)
Current	2.46 (1.58; 3.82)	2.52 (1.61; 3.93)		2.17 (1.40; 3.37)	3.78 (2.22; 6.44)	

	Cardia GC (*N* = 272/4087)			Cardia GC (*N* = 188/2978)		
	**Normal weight**	**Overweight**	**RERI**	**Normal weight**	**Obesity**	**RERI**
	OR (95% CI)	OR (95% CI)	(95% CI)	OR (95% CI)	OR (95% CI)	(95% CI)
Smoking status						
Never	1.00	2.06 (1.39; 3.05)	–0.66 (–1.85; 0.53)	1.00	2.24 (1.43; 3.49)	–0.77 (–2.07; 0.53)
Former	2.41 (1.53; 3.80)	2.81 (1.81; 4.35)		1.85 (1.17; 2.92)	2.32 (1.40; 3.84)	

ORs are adjusted for age, sex, study, alcohol-drinking status, and socioeconomic status.

**Table 4.** Interaction analysis between BMI (overweight or obesity vs normal weight) and drinking status (low or intermediate or high vs never drinker) in cardia GC.

	Cardia GC (*N* = 201/2870)			Cardia GC (*N* = 133/2142)		
	Normal weight	Overweight	RERI	Normal weight	Obesity	RERI
	OR (95% CI)	OR (95% CI)	(95% CI)	OR (95% CI)	OR (95% CI)	(95% CI)
Drinking status						
Never	1.00	2.08 (1.42; 3.06)	–1.13 (–2.68; 0.42)	1.00	1.72 (1.04; 2.85)	0.58 (–1.25; 2.41)
Low	2.52 (1.38; 4.58)	2.47 (1.39; 4.36)		1.87 (1.01; 3.47)	3.17 (1.61; 6.26)	

	Cardia GC (*N* = 202/2585)			Cardia GC (*N* = 132/1890)		
	Normal weight	Overweight	RERI	Normal weight	Obesity	RERI
	OR (95% CI)	OR (95% CI)	(95% CI)	OR (95% CI)	OR (95% CI)	(95% CI)
Drinking status						
Never	1.00	2.09 (1.42; 3.08)	–0.07 (–0.62; 0.48)	1.00	1.68 (1.02; 2.77)	0.44 (–0.16; 1.05)
Intermediate	1.27 (0.94; 1.73)	2.29 (1.54; 3.41)		1.17 (0.85; 1.61)	2.29 (1.42; 3.71)	

	Cardia GC (*N* = 244/2099)			Cardia GC (*N* = 171/1586)		
	Normal weight	Overweight	RERI	Normal weight	Obesity	RERI
	OR (95% CI)	OR (95% CI)	(95% CI)	OR (95% CI)	OR (95% CI)	(95% CI)
Drinking status						
Never	1.00	2.04 (1.39; 3.00)	–0.29 (–0.61; 0.03)	1.00	1.69 (1.03; 2.78)	–0.02 (–0.35; 0.31)
High	1.64 (1.36; 1.96)	2.39 (1.64; 3.48)		1.51 (1.25; 1.83)	2.18 (1.38; 3.45)	

ORs are adjusted for age, sex, study, smoking status, and socioeconomic status.

**Figure 2.**
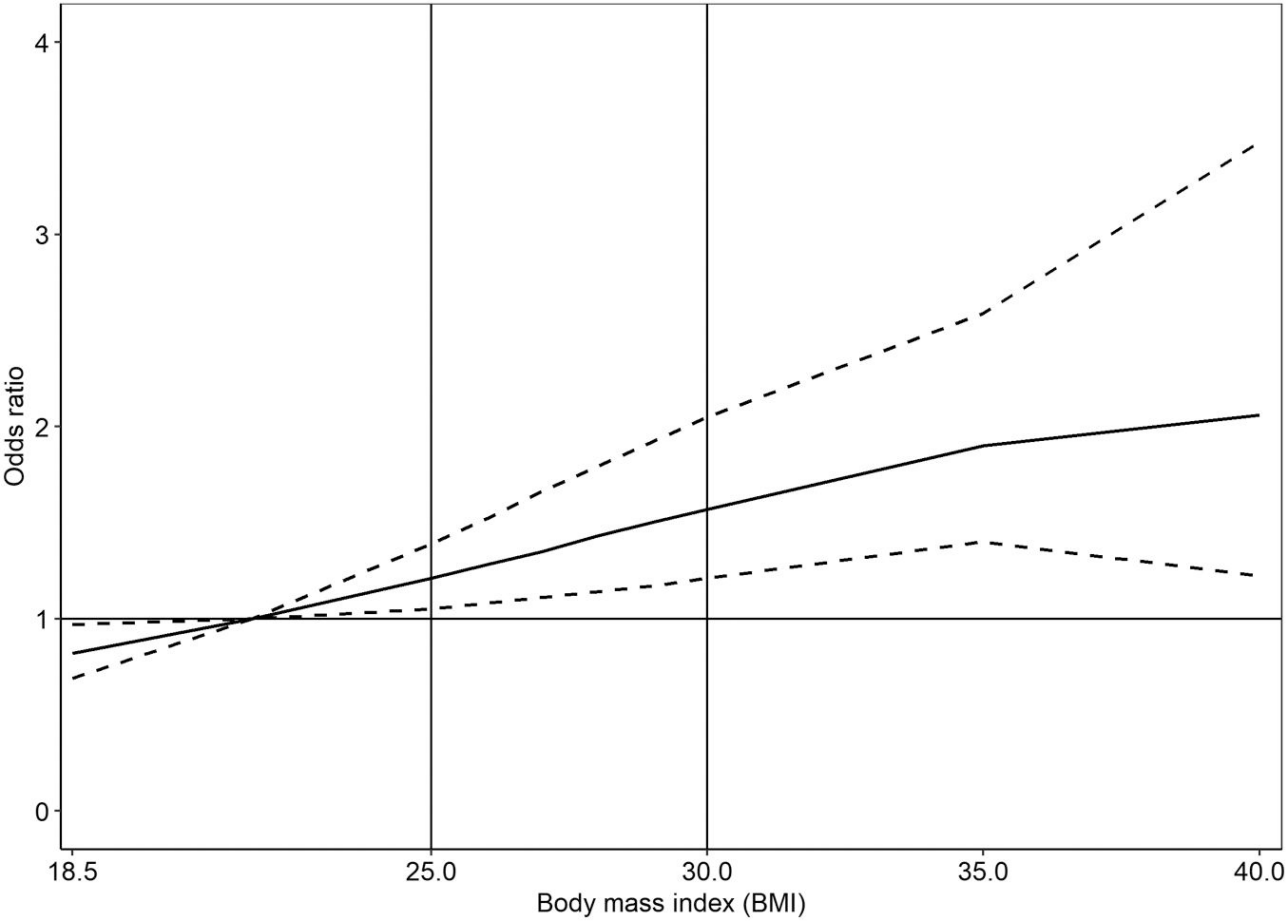
Dose–response relationship between BMI and cardia GC fitted by using one-stage logistic mixed-effects model with a second-order (powers 2 and 3) fractional polynomial (*N* = 7099). Solid black line: adjusted OR; dashed black line: 95% CI.

The relationship between the value of BMI and cardia GC is depicted in Fig. 2. The risk of cardia GC increases with higher BMI values, ranging from a null effect when considering the average value of the normal weight category (BMI = 21.75) as a reference to an OR of 2.06 (95% CI 1.22–3.48) for a BMI of 40 (Table 5).

**Table 5.** Results of the dose–response analysis on BMI and cardia GC fitted by using one-stage logistic mixed-effects model with fractional polynomial (*N* = 7099).

BMI values	OR (95% CI)
18.5	0.82 (0.69–0.97)
21.75 (ref.)	1.00
25	1.21 (1.05–1.39)
26	1.28 (1.08–1.52)
27	1.35 (1.11–1.66)
28	1.43 (1.14–1.79)
29	1.50 (1.17–1.92)
30	1.57 (1.21–2.05)
35	1.90 (1.40–2.59)
≥40	2.06 (1.22–3.48)

ORs are adjusted for age, sex, study, smoking status, alcohol-drinking status, and socioeconomic status.

In additional analysis on Obesity Class I and II/III (1290 and 361 subjects, respectively), the risk of cardia GC increases with higher obesity classes: Obesity Class I has an OR of 1.53 (95% CI 1.14–2.04) and Obesity Class II/III has an OR of 1.81 (95% CI 1.15–2.84) (Supplementary Figure 3).

The analyses conducted by using only studies that enrolled population-based controls (10-Iran, 21-Spain, and 32-USA) report an association between increased BMI and the risk of cardia GC, with ORs of 1.62 (95% CI 1.10–2.39) for overweight individuals and 1.65 (95% CI 1.09–2.48) for obese individuals, whereas the results do not change for the risk of non-cardia GC (Supplementary Figure 4). Similarly, the analysis conducted on six studies and including adjustment for fruit-and-vegetables intake and salt intake confirmed the association with cardia GC, with similar results: OR 1.34 (95% CI 1.01–1.80) for overweight and OR 1.51 (95% CI 1.06–2.14) for obesity.

## Discussion

Our pooled analysis of seven case–control studies from the international StoP Consortium showed that obese subjects have a around 60% increased risk of cardia GC. When the analysis was restricted to the four studies enrolling population-based controls, the risk of cardia GC increased also among overweight individuals. The dose–response meta-analysis confirmed a risk gradient across the range of BMI values. Conversely, the risk of non-cardia GC does not seem to be affected by the BMI.

The association between an increased BMI and the risk of cardia GC is plausible and aligns with previous meta-analyses published in the literature [11–13, 18]. Obesity may exacerbate gastroesophageal reflux disease, potentially leading to Barrett’s esophagus, a known precursor to esophageal adenocarcinoma and cardia GC [35, 36]. Additionally, the accumulation of adipose tissue in obesity is thought to increase the production of various endogenous hormones such as sex steroids, insulin, and insulin-like growth factor-1 [37]. These hormonal changes can promote cellular proliferation and hinder apoptotic processes, thereby facilitating the growth of preneoplastic and neoplastic cells. Furthermore, obesity is characterized as a pro-inflammatory state, associated with elevated levels of pro-inflammatory cytokines, including tumor necrosis factor and interleukin-6 [10]. These cytokines contribute to cancer development, further supporting the connection between obesity and cardia GC. Obese individuals experience an extended esophageal transit time, which implies that the esophageal mucosa is exposed to food (which may contain potentially carcinogenic components) for a longer duration [38]. This prolonged contact time could potentially facilitate the development of cancer. To better investigate this hypothesis, it would be helpful to study the association with abdominal fat or waist circumference. However, these data were not collected by enough studies in the consortium, within the predefined criteria for this investigation, to perform analyses. Supporting this hypothesis, a meta-analysis of six cohort studies conducted by Du *et al.* published in 2017 did indeed observe an association between waist circumference and the risk of both cardia GC and esophageal adenocarcinoma [39].

The observed dose–response relationship between BMI and cardia GC risk substantiates the hypothesis that excess body weight constitutes a risk factor for the development of GC and it is consistent with previous studies [13, 40, 41]. This consistent escalation in risk with increasing BMI may reflect complex interactions between metabolic dysfunction, chronic inflammation, and hormonal imbalances, all of which are exacerbated by higher adiposity levels and have been implicated in oncogenesis [37, 42].

In our study, by restricting the analysis to only studies that employed population-based controls, we observed an increased risk of cardia GC also among overweight subjects. This suggests that the use of hospital-based controls may have introduced selection bias, as these controls were likely affected by clinical conditions associated with an increased BMI. Indeed, a high BMI is a known risk factor for numerous diseases, particularly cardiovascular diseases and cancer [43, 44], and therefore it is necessary to take this into account when synthesizing the evidence.

Our stratified analyses revealed that overweight individuals had a significantly increased risk of cardia GC among nonsmokers (OR 1.92, 95% CI 1.29–2.85) and nondrinkers (OR 2.20, 95% CI 1.48–3.27), suggesting that the impact of excess body weight may be more pronounced in the absence of other major carcinogenic exposures. This finding supports the hypothesis that smoking and alcohol consumption, which are well-established risk factors for GC, may obscure or attenuate the relationship between BMI and cancer risk, a pattern also observed in previous studies conducted in Asian populations [45, 46]. To the best of our knowledge, the additive interaction between smoking and BMI, as well as between alcohol and BMI, on GC risk has not been explored in the literature before. The lack of a significant positive or negative additive interaction may reflect overlapping biological pathways or a ceiling effect among individuals already exposed to potent carcinogens.

Our study does not support an association between high BMI and non-cardia GC. These findings were confirmed in all the sensitivity analysis performed and are in line with previously published literature [14, 43, 47]. This aligns with the understanding that the etiological mechanisms of non-cardia GC and cardia GC are likely different [6, 7]. Non-cardia cancers are typically caused by chronic gastritis, an inflammation of the stomach lining, which can be induced by a range of environmental factors [48, 49]. Additionally, the lack of association, and in some instances even an inverse association, between BMI and non-cardia GC could be attributed to the prevalence of malnutrition among smokers and alcohol drinkers, who are at an increased risk for this cancer, i.e. reverse causation [44].

Our study has several strengths, including the large sample size, the pooling of data from multiple studies, and the sensitivity analyses performed. The pooled-analysis approach enhances statistical power, allows the harmonization of variable definitions across studies, and enables a more comprehensive assessment of potential confounders, effect modifiers, and subgroup-specific associations [50]. Moreover, our study includes data from European populations, which are typically underrepresented in GC research that predominantly focuses on Asian populations. However, there are some limitations. Firstly, our analysis was based on case–control studies, which relied on self-reported data on weight before symptoms of the disease and other variables such as smoking, making them susceptible to recall bias. This concern may be particularly relevant for studies that report the maximum weight recorded during an individual’s lifetime. However, we utilized the BMI categories defined by the WHO. As a substantial change in BMI would be required to alter the classification into a different category, this approach helps to minimize the risk of misclassification related to recall bias and its potential impact on our findings.

Secondly, although we restricted our analysis to studies that provided weight data from ≥1 year prior to diagnosis to mitigate the potential influence of the disease on weight, we acknowledge that GC is often diagnosed at an advanced stage. Consequently, the recorded weight 1 year before diagnosis may have been partially influenced by the disease onset. However, six out of seven studies recorded weight from ≥3 years before enrollment and any potential influence of the disease on weight would likely lead to an underestimation of the effect of BMI on cancer risk. Third, BMI neither captures body fat distribution nor differentiates between lean mass and fat mass. It is also important to note that residual confounding may be present due to unmeasured factors in the studies included in the meta-analysis, such as air pollution or other dietary factors, whose role in the etiology of GC is uncertain.

## Conclusions

Our study provides evidence that overweight and obesity are associated with an increased risk of cardia GC, whereas they show no association with the risk of non-cardia GC. However, certain questions remain regarding potential effect modifiers and underlying mechanisms that warrant further epidemiological investigation. These findings contribute to a better understanding of GC etiology and may inform targeted primary and secondary preventive interventions for high-risk populations.

## Ethics approval

The participating studies were conducted in accordance with applicable laws, regulations, and guidelines for the protection of human patients. In addition, the StoP Project received ethical approval from the University of Milan Review Board (reference 19/15, 1 April 2015).

## Supplementary Material

dyaf160_Supplementary_Data

## Data Availability

Data used for this study are available upon formal request from the corresponding author, after approval of the participating study centers of the StoP Project.
