# Supplementary material for: Body mass index and gastric cancer risk: results from the Stomach Cancer Pooling Project Consortium
Source: Int J Epidemiol. 2025 Sep 28;54(5):dyaf160. doi: 10.1093/ije/dyaf160 (PMC12476909; doi:10.1093/ije/dyaf160)
Supplement: dyaf160_Supplementary_Data [file dyaf160_supplementary_data.docx]

**Supplementary material**

**BMI and gastric cancer risk: a pooled study within the Stomach cancer Pooling (StoP) Project**

[StoP Project Methodology 2](#_Toc200979662)

[Supplementary Table S1. Characteristics of individual studies of the StoP consortium included in the analysis. 3](#_Toc200979663)

[Supplementary Table S2a. Main characteristics of cardia and non-cardia gastric cancer cases and controls in the Stomach cancer Pooling (StoP) Project consortium studies included in the analysis – 03-Italy, 09-Russia, 10-Iran and 16-USA. 4](#_Toc200979664)

[Supplementary Table S2b. Main characteristics of cardia and non-cardia gastric cancer cases and controls in the Stomach cancer Pooling (StoP) Project consortium studies included in the analysis – 21-Spain, 23-Spain and 32-USA 6](#_Toc200979665)

[Supplementary Figure S1. Galbraith plot examining heterogeneity between studies in the association between BMI overweight and risk of non-cardia gastric cancer. 8](#_Toc200979666)

[Supplementary Figure S2. Study-specific and adjusted pooled odds ratios (ORs) and corresponding 95% confidence intervals (CIs) for the association between BMI and cardia gastric cancer risk, and separately non-cardia gastric cancer risk using the one-stage approach, and comparison with the two-stage results. 9](#_Toc200979667)

[Supplementary Figure S3. Odds ratios (ORs) specific to each study and adjusted pooled ORs with corresponding 95% confidence intervals (CIs) for the association between cardia gastric cancer risk and BMI categories (overweight, obesity class I and II/III). 10](#_Toc200979668)

[Supplementary Figure S4. Odds ratios (ORs) specific to each study and adjusted pooled ORs with corresponding 95% confidence intervals (CIs) for the association between cardia gastric cancer risk and BMI categories (overweight and obese individuals) using only population-based controls. 11](#_Toc200979669)

# **StoP Project Methodology**

The StoP Project, initiated in 2012, is an epidemiological consortium aiming at examining the role of lifestyle and genetic factors in the etiology of GC through pooled analyses of individual-level data from previously conducted observational studies.  Inclusion criteria for study participation in the Project are case–control study design, including nested case–control within cohort studies, and inclusion of at least 80 cases of incident, histologically confirmed gastric cancer (including both cardia and noncardia locations). The Project involves central collection, validation, and harmonization of the original data.

This study is based on the third release (v3.1) of the StoP Project dataset, which includes 34 case-control studies—or case-control analyses nested within cohorts—from 14 countries, totaling 13,121 gastric cancer cases and 31,420 controls. GC cases were incident and histologically confirmed at diagnosis, while controls were either population-based or hospital-based cancer-free individuals, selected to match cases in time and geography.

For each study, investigators completed a description form and provided contact information. Participating studies shared either the full dataset or a core set of standardized variables (e.g., age, sex, education, smoking, dietary factors, *H. pylori* markers), along with original questionnaires and supporting materials such as codebooks. All datasets were harmonized at the coordinating center (University of Milan) using a pre-specified format. Data were organized into thematic sections and harmonized through the creation of project-specific codebooks and recoding rules. A unique identifier was assigned to each participant to ensure traceability and consistency across the integrated dataset.

To ensure data quality, completeness and consistency checks were centrally performed using dedicated programs to detect implausible values, inconsistencies, and missing data patterns. Frequency distributions, summary statistics, and feedback reports were shared with each study for verification and correction. Ethical approval for the StoP Project was granted by the University of Milan Review Board (Approval No. 19/15, dated April 1, 2015).

# **Supplementary Table S1.** Characteristics of individual studies of the StoP consortium included in the analysis.

| **Study** | **Recruitment period** | **Type of controls** | **Type of matching (Matching factors)** | **Reported BMI in cases** | **Reference** |
| --- | --- | --- | --- | --- | --- |
| 03-Italy | 1997-2007 | Hospital based | Frequency (Age, Sex) | Max* | Lucenteforte et al., 2008 ^40^ |
| 09-Russia | 1996-1997 | Hospital based | None | Max* | Zaridze et al., 1999 ^40^ |
| 10-Iran | 2004-2005 | Population based | Frequency (Age, Sex) | 10 years** | Pourfarzi et al., 2009 |
| 16-USA | 1980-1990 | Hospital based | Frequency (Age, Sex) | 5 years** | Muscat J. (Unpublished data) |
| 21-Spain | 2008-2012 | Population based | Frequency (Age, Sex, Recruitment area) | 1 year** | Castaño-Vinyals et al., 2015 |
| 23-Spain | 1995-1999 | Hospital based | Frequency (Age, Sex, Recruitment area) | 5 years** | Santibanez et al., 2012 |
| 32-USA | 1988-1993 | Population based | Frequency (Age, Sex, Recruitment area) | 3-8 years** | Ward et al., 1997 |

* Maximal in lifetime

** Years before diagnosis

# **Supplementary Table S2a.** Main characteristics of cardia and non-cardia gastric cancer cases and controls in the Stomach cancer Pooling (StoP) Project consortium studies included in the analysis – 03-Italy, 09-Russia, 10-Iran and 16-USA.

|  | **03-Italy** | | | | **09-Russia** | | | | **10-Iran** | | | | **16-USA** | | | |
| --- | --- | --- | --- | --- | --- | --- | --- | --- | --- | --- | --- | --- | --- | --- | --- | --- |
|  | **Control** | **Cardia** | **Non-cardia** | **Total** | **Control** | **Cardia** | **Non-cardia** | **Total** | **Control** | **Cardia** | **Non-cardia** | **Total** | **Control** | **Cardia** | **Non-cardia** | **Total** |
|  | **N=523** | **N=10** | **N=91** | **N=624** | **N=504** | **N=78** | **N=165** | **N=747** | **N=390** | **N=114** | **N=81** | **N=585** | **N=1322** | **N=158** | **N=2** | **N=1482** |
| **Sex, n (%)** |  |  |  |  |  |  |  |  |  |  |  |  |  |  |  |  |
| Male | 277 (53.0) | 7 (70.0) | 50 (54.9) | 334 (53.4) | 239 (47.4) | 52 (66.7) | 78 (47.3) | 369 (49.4) | 263 (67.4) | 73 (64.0) | 64 (79 .0) | 400 (68.4) | 1082 (81.8) | 136 (86.1) | 0 (0.0) | 1218 (82.2) |
| Female | 246 (47.0) | 3 (30.0) | 41 (45.1) | 290 (46.4) | 265 (52.6) | 26 (33.3) | 87 (52.7) | 378 (50.6) | 127 (32.6) | 41 (36.0) | 17 (21.0) | 185 (31.6) | 240 (18.2) | 22 (13.9) | 2 (100) | 264 (17.8) |
| **Age (years), n (%)** |  |  |  |  |  |  |  |  |  |  |  |  |  |  |  |  |
| <65 | 290 (55.4) | 3 (30.0) | 62 (68.1) | 355 (56.8) | 386 (76.6) | 41 (52.6) | 86 (52.1) | 513 (68.7) | 157 (40.3) | 45 (39.5) | 31 (38.3) | 233 (39.8) | 833 (63.0) | 97 (61.4) | 0 (0.0) | 930 (62.8) |
| ≥65 | 233 (44.6) | 7 (70.0) | 29 (31.9) | 269 (43.0) | 118 (23.4) | 37 (47.4) | 79 (47.9) | 234 (31.3) | 233 (59.7) | 69 (60.5) | 50 (61.7) | 352 (60.2) | 489 (37.0) | 61 (38.6) | 2 (100) | 552 (37.2) |
| **BMI, n (%)** |  |  |  |  |  |  |  |  |  |  |  |  |  |  |  |  |
| Underweight | 5 (1.0) | 0 (0.0) | 2 (2.2) | 7 (1.1) | 0 (0.0) | 0 (0.0) | 0 (0.0) | 0 (0.0) | 9 (2.3) | 0 (0.0) | 2 (2.5) | 11 (1.9) | 16 (1.2) | 1 (0.6) | 0 (0.0) | 17 (1.1) |
| Normal weight | 163 (31.2) | 2 (20.0) | 36 (39.6) | 201 (32.2) | 130 (25.8) | 19 (24.4) | 45 (27.3) | 194 (26.0) | 190 (48.7) | 38 (33.3) | 28 (34.6) | 256 (43.8) | 468 (35.4) | 53 (33.5) | 0 (0.0) | 521 (35.2) |
| Overweight | 235 (44.9) | 3 (30.0) | 34 (37.4) | 272 (43.5) | 202 (40.1) | 33 (42.3) | 76 (46.1) | 311 (41.6) | 139 (35.6) | 60 (52.6) | 44 (54.3) | 243 (41.5) | 613 (46.4) | 68 (43.0) | 2 (100) | 683 (46.1) |
| Obesity | 120 (22.9) | 5 (50.0) | 19 (20.9) | 144 (23.0) | 172 (34.1) | 26 (33.3) | 44 (26.7) | 242 (32.4) | 52 (13.3) | 16 (14.0) | 7 (8.6) | 75 (12.8) | 225 (17.0) | 36 (22.8) | 0 (0.0) | 261 (17.6) |
| **Socioeconomic status, n (%)** |  |  |  |  |  |  |  |  |  |  |  |  |  |  |  |  |
| Low | 221 (42.3) | 4 (40.0) | 31 (34.1) | 256 (41.0) | 19 (3.8) | 2 (2.6) | 8 (4.8) | 29 (3.9) | 285 (73.1) | 104 (91.2) | 65 (80.2) | 454 (77.6) | 131 (9.9) | 8 (5.1) | 0 (0.0) | 139 (9.4) |
| Intermediate | 169(32.3) | 5 (50.0) | 37 (40.6) | 211 (33.8) | 447 (88.7) | 68 (87.2) | 137 (83.0) | 652 (87.3) | 79 (20.3) | 5 (4.4) | 14 (17.3) | 98 (16.8) | 828 (62.6) | 83 (52.5) | 1 (50.0) | 912 (61.5) |
| High | 130 (24.9) | 1 (10.0) | 23 (25.3) | 154 (24.7) | 33 (6.5) | 8 (10.2) | 18 (10.9) | 59 (7.9) | 26 (6.7) | 5 (4.4) | 2 (2.5) | 33 (5.6) | 362 (27.4) | 67 (42.4) | 1 (50.0) | 430 (29.0) |
| Missing | 3 (0.6) | - | - | 3 (0.5) | 5 (1.0) | - | 2 (1.2) | 7 (1.9) | - | - | - | - | 1 (0.1) | - | - | 1 (0.1) |
| **Smoking status, n (%)** |  |  |  |  |  |  |  |  |  |  |  |  |  |  |  |  |
| Never smoker | 243 (46.5) | 3 (30.0) | 36 (39.6) | 282 (45.1) | 289 (57.3) | 34 (43.6) | 104 (63.0) | 427 (57.2) | 249 (63.8) | 76 (66.7) | 44 (54.3) | 369 (63.1) | 462 (34.9) | 37 (23.4) | 1 (50.0) | 500 (33.7) |
| Former smoker | 159 (30.4) | 3 (30.0) | 25 (27.5) | 187 (29.9) | 78 (15.5) | 11 (14.1) | 21 (12.7) | 110 (14.7) | 42 (10.8) | 12 (10.5) | 9 (11.1) | 63 (10.8) | 573 (43.3) | 77 (48.7) | 1 (50.0) | 651 (43.9) |
| Current smoker | 121 (23.1) | 4 (40.0) | 30 (33.0) | 155 (24.8) | 137 (27.2) | 32 (41.0) | 40 (24.2) | 209 (28.0) | 99 (25.4) | 26 (22.8) | 28 (34.6) | 153 (26.2) | 287 (21.7) | 44 (27.8) | 0 (0.0) | 331 (22.3) |
| Missing | - | - | - | - | - | 1 (0.3) | - | 1 (0.1) | - | - | - | - | - | - | - | - |
| **Alcohol drinking status, n (%)** |  |  |  |  |  |  |  |  |  |  |  |  |  |  |  |  |
| Never | 116 (22.2) | 1 (10.0) | 14 (15.4) | 131 (21.0) | 125 (24.8) | 15 (19.2) | 43 (26.1) | 183 (24.5) | 383 (98.2) | 113 (99.1) | 77 (95.1) | 573 (97.9) | 332 (25.1) | 21 (13.3) | 1 (50.0) | 354 (23.9) |
| Low | 159 (30.4) | 3 (30.0) | 28 (30.8) | 190 (30.4) | 30 (6.0) | 2 (2.6) | 5 (3.0) | 37 (5.0) | 6 (1.5) | 0 (0.0) | 1 (1.2) | 7 (1.2) | 322 (24.4) | 40 (25.3) | 1 (50.0) | 363 (24.5) |
| Intermediate | 183 (35.0) | 1 (10.0) | 35 (38.5) | 219 (35.0) | 125 (24.8) | 18 (23.1) | 41 (24.8) | 184 (24.6) | 1 (0.3) | 1 (0.9) | 2 (2.5) | 4 (0.7) | 233 (17.6) | 24 (15.2) | 0 (0.0) | 257 (17.3) |
| High | 65 (12.4) | 5 (50.0) | 14 (15.4) | 84 (13.4) | 213 (42.3) | 42 (53.8) | 70 (42.4) | 325 (43.5) | 0 (0.0) | 0 (0.0) | 1 (1.2) | 1 (0.2) | 361 (27.3) | 70 (44.3) | 0 (0.0) | 431 (29.1) |
| Missing | - | - | - | - | 11 (2.1) | 1 (0.3) | 6 (3.9) | 18 (2.4) | - | - | - | - | 74 (5.6) | 3 (1.9) | - | 77 (5.2) |
| **Fruit and vegetables intake, n(%)** |  |  |  |  |  |  |  |  |  |  |  |  |  |  |  |  |
| Low | 168 (32.1) | 3 (30.0) | 21 (23.1) | 192 (30.8) | 44 (8.7) | 6 (7.7) | 9 (5.5) | 59 (7.9) | 44 (11.3) | 2 (1.8) | 4 (4.9) | 50 (8.5) | 0 (0.0) | 0 (0.0) | 0 (0.0) | 0 (0.0) |
| Intermediate | 171 (32.7) | 4 (40.0) | 43 (47.3) | 218 (34.9) | 147 (29.2) | 22 (28.2) | 52 (31.5) | 221 (29.6) | 123 (31.5) | 47 (41.2) | 35 (43.2) | 205 (35.0) | 0 (0.0) | 0 (0.0) | 0 (0.0) | 0 (0.0) |
| High | 184 (35.2) | 3 (30.0) | 27 (19.7) | 214 (34.3) | 309 (61.3) | 50 (64.1) | 104 (63.0) | 463 (62.0) | 222 (56.9) | 65 (57.0) | 41 (50.6) | 328 (56.1) | 0 (0.0) | 0 (0.0) | 0 (0.0) | 0 (0.0) |
| Missing | - | - | - | - | 4 (0.8) | 0 (0.0) | 0 (0.0) | 4 (0.5) | 1 (0.3) | 0 (0.0) | 1 (1.2) | 2 (0.3) | 1322 (100) | 158 (100) | 2 (100) | 1482 (100) |
| **Salt intake, n (%)** |  |  |  |  |  |  |  |  |  |  |  |  |  |  |  |  |
| Low | 363 (69.4) | 10 (100.0) | 71 (78.0) | 444 (71.2) | 148 (29.4) | 18 (23.1) | 35 (21.2) | 201 (26.9) | 297 (76.2) | 49 (43.0) | 28 (34.6) | 374 (63.9) | 0 (0.0) | 0 (0.0) | 0 (0.0) | 0 (0.0) |
| Intermediate | 108 (20.7) | 0 (0.0) | 15 (16.5) | 123 (19.7) | 326 (64.7) | 56 (71.8) | 125 (75.8) | 507 (67.9) | 65 (16.7) | 45 (39.5) | 34 (42.0) | 144 (24.6) | 0 (0.0) | 0 (0.0) | 0 (0.0) | 0 (0.0) |
| High | 50 (9.6) | 0 (0.0) | 5 (5.5) | 55 (8.8) | 30 (6.0) | 4 (5.1) | 5 (3.0) | 39 (5.2) | 25 (6.4) | 20 (17.5) | 18 (22.2) | 63 (10.8) | 0 (0.0) | 0 (0.0) | 0 (0.0) | 0 (0.0) |
| missing | 2 (0.4) | 0 (0.0) | 0 (0.0) | 2 (0.3) | - | - | - | - | 3 (0.8) | 0 (0.0) | 1 (1.2) | 4 (0.7) | 1322 (100) | 158 (100) | 2 (100) | 1482 (100) |
| **History of diabetes, n (%)** |  |  |  |  |  |  |  |  |  |  |  |  |  |  |  |  |
| No | 485 (92.7) | 8 (80.0) | 88 (96.7) | 581 (93.0) | 462 (91.7) | 72 (92.3) | 150 (90.9) | 684 (91.6) | 0 (0.0) | 0 (0.0) | 0 (0.0) | 0 (0.0) | 1186 (89.7) | 143 (90.5) | 1 (50.0) | 1330 (89.7) |
| Yes | 38 (7.3) | 2 (20.0) | 3 (3.3) | 43 (6.9) | 42 (8.3) | 6 (7.7) | 15 (9.1) | 63 (8.4) | 0 (0.0) | 0 (0.0) | 0 (0.0) | 0 (0.0) | 136 (10.3) | 15 (9.5) | 1 (50.0) | 152 (10.3) |
| Missing | - | - | - | - | - | - | - | - | 390 (100) | 114 (100) | 81 (100) | 585 (100) | - | - | - | - |
| ***H. pylori* infection status, n(%)** |  |  |  |  |  |  |  |  |  |  |  |  |  |  |  |  |
| Negative | 0 (0.0) | 0 (0.0) | 0 (0.0) | 0 (0.0) | 192 (38.1) | 40 (51.3) | 61 (37.0) | 293 (39.2) | 107 (27.4) | 22 (19.3) | 12 (14.8) | 141 (24.1) | 0 (0.0) | 0 (0.0) | 0 (0.0) | 0 (0.0) |
| Positive | 0 (0.0) | 0 (0.0) | 0 (0.0) | 0 (0.0) | 175 (34.7) | 23 (29.5) | 65 (39.4) | 263 (35.2) | 267 (68.5) | 82 (71.9) | 59 (72.8) | 408 (69.7) | 0 (0.0) | 0 (0.0) | 0 (0.0) | 0 (0.0) |
| Missing | 523 (100) | 10 (100) | 91 (100) | 624 (100) | 137 (27.2) | 15 (19.2) | 39 (33.6) | 191 (25.6) | 16 (4.1) | 10 (8.8) | 10 (12.4) | 36 (6.2) | 1322 (100) | 158 (100) | 2 (100) | 1482 (100) |
| **Family history of GC, n (%)** |  |  |  |  |  |  |  |  |  |  |  |  |  |  |  |  |
| No | 492 (94.1) | 10 (100) | 79 (86.8) | 581 (93.0) | 423 (83.9) | 63 (80.8) | 128 (77.6) | 614 (82.2) | 366 (93.8) | 102 (89.5) | 66 (81.5) | 534 (91.3) | 0 (0.0) | 0 (0.0) | 0 (0.0) | 0 (0.0) |
| Yes | 31 (5.9) | 0 (0.0) | 12 (13.2) | 43 (6.9) | 67 (13.3) | 12 (15.4) | 30 (18.2) | 109 (14.6) | 24 (6.2) | 12 (10.5) | 15 (18.5) | 51 (8.7) | 0 (0.0) | 0 (0.0) | 0 (0.0) | 0 (0.0) |
| Missing | - | - | - | - | 14 (2.8) | 3 (3.8) | 7 (3.8) | 24 (3.2) | - | - | - | - | 1322 (100) | 158 (100) | 2 (100) | 1482 (100) |
| **Histological type, n %** |  |  |  |  |  |  |  |  |  |  |  |  |  |  |  |  |
| Intestinal | - | 3 (30.0) | 13 (14.3) | 16 (2.6) | - | 25 (32.1) | 40 (24.2) | 65 (8.7) | - | 64 (56.1) | 50 (61.7) | 114 (19.5) | - | 0 (0.0) | 0 (0.0) | 0 (0.0) |
| Diffuse | - | 0 (0.0) | 26 (28.6) | 26 (4.2) | - | 23 (29.5) | 43 (26.1) | 66 (8.8) | - | 25 (21.9) | 28 (34.6) | 53 (9.1) | - | 0 (0.0) | 0 (0.0) | 0 (0.0) |
| Mixed/Undifferentiated | - | 4 (40.0) | 35 (38.5) | 39 (6.2) | - | 30 (38.5) | 82 (49.7) | 112 (15.0) | - | 25 (21.9) | 3 (3.7) | 28 (4.8) | - | 158 (100) | 2 (100) | 160 (10.8) |
| Missing | - | 3 (30.0) | 17 (18.7) | 20 (3.2) | - | - | - | - | - | - | - | - | - | - | - | - |

# **Supplementary Table S2b.** Main characteristics of cardia and non-cardia gastric cancer cases and controls in the Stomach cancer Pooling (StoP) Project consortium studies included in the analysis – 21-Spain, 23-Spain and 32-USA

|  | **21-Spain** | | | | **23-Spain** | | | | **32-USA** | | | |
| --- | --- | --- | --- | --- | --- | --- | --- | --- | --- | --- | --- | --- |
|  | **Control** | **Cardia** | **Non-cardia** | **Total** | **Control** | **Cardia** | **Non-cardia** | **Total** | **Control** | **Cardia** | **Non-cardia** | **Total** |
|  | **N=3056** | **N=91** | **N=311** | **N=3458** | **(N=397)** | **(N=28)** | **(N=213)** | **(N=638)** | **(N=479)** | **(N=32)** | **(N=104)** | **(N=615)** |
| **Sex, n (%)** |  |  |  |  |  |  |  |  |  |  |  |  |
| Male | 1713 (56.1) | 81 (89.0) | 190 (61.1) | 1984 (57.4) | 264 (66.5) | 24 (85.7) | 146 (68.5) | 434 (68.0) | 269 (56.2) | 25 (78.1) | 53 (51.0) | 347 (56.4) |
| Female | 1343 (43.9) | 10 (11.0) | 121 (38.9) | 1474 (42.6) | 133 (33.5) | 4 (14.3) | 67 (31.5) | 204 (32.0) | 210 (43.8) | 7 (21.9) | 51 (49.0) | 268 (43.6) |
| **Age (years), n (%)** |  |  |  |  |  |  |  |  |  |  |  |  |
| <65 | 1447 (47.3) | 43 (47.3) | 110 (35.4) | 1600 (46.3) | 207 (52.1) | 11 (39.3) | 92 (43.2) | 310 (48.6) | 173 (36.1) | 10 (31.3) | 15 (14.4) | 198 (32.2) |
| ≥65 | 1609 (52.7) | 48 (52.7) | 201 (64.6) | 1858 (53.7) | 190 (47.9) | 17 (60.7) | 121 (56.8) | 328 (51.4) | 306 (63.9) | 22 (68.8) | 89 (85.6) | 417 (67.8) |
| **BMI, n (%)** |  |  |  |  |  |  |  |  |  |  |  |  |
| Underweight | 34 (1.1) | 1 (1.1) | 2 (0.6) | 37 (1.1) | 5 (1.3) | 0 (0.0) | 3 (1.4) | 8 (1.3) | 12 (2.5) | 0 (0.0) | 2 (1.9) | 14 (2.3) |
| Normal weight | 1093 (35.8) | 21 (23.1) | 96 (30.9) | 1210 (35.0) | 128 (32.2) | 10 (35.7) | 88 (41.3) | 226 (35.4) | 245 (51.1) | 12 (37.5) | 44 (42.3) | 301 (48.9) |
| Overweight | 1291 (42.2) | 42 (46.2) | 145 (46.6) | 1478 (42.7) | 176 (44.3) | 11 (39.3) | 87 (40.8) | 274 (42.9) | 171 (35.7) | 14 (43.8) | 49 (47.1) | 234 (38.0) |
| Obesity | 638 (20.9) | 27 (29.7) | 68 (21.9) | 733 (21.2) | 88 (22.2) | 7 (25.0) | 35 (16.4) | 130 (20.4) | 51 (10.6) | 6 (18.8) | 9 (8.7) | 66 (10.7) |
| **Socioeconomic status, n (%)** |  |  |  |  |  |  |  |  |  |  |  |  |
| Low | 1523 (49.8) | 57 (62.6) | 220 (70.7) | 1800 (52.1) | 360 (90.7) | 26 (92.9) | 196 (92.0) | 582 (91.2) | 21 (4.4) | 3 (9.4) | 11 (10.6) | 35 (5.7) |
| Intermediate | 881 (28.8) | 22 (24.2) | 60 (19.3) | 963 (27.8) | 30 (7.6) | 2 (7.1) | 13 (6.1) | 45 (7.1) | 262 (54.7) | 18 (56.3) | 69 (66.3) | 349 (56.7) |
| High | 652 (21.3) | 12 (13.2) | 31(10.0) | 695 (20.1) | 7 (1.8) | 0 (0.0) | 4 (1.9) | 11 (1.7) | 166 (34.6) | 11 (34.4) | 24 (23.1) | 201 (32.7) |
| Missing | - | - | - | - | - | - | - | - | ­30 (6.3) | - | - | 30 (4.9) |
| **Smoking status, n (%)** |  |  |  |  |  |  |  |  |  |  |  |  |
| Never smoker | 1393 (45.6) | 24 (26.4) | 147 (47.3) | 1564 (45.2) | 188 (47.4) | 9 (32.1) | 91 (42.7) | 288 (45.1) | 218 (45.5) | 7 (21.9) | 59 (56.7) | 284 (46.2) |
| Former smoker | 1081 (35.4) | 38 (41.8) | 103 (33.1) | 1222 (35.3) | 107 (27.0) | 9 (32.1) | 43 (20.2) | 159 (24.9) | 182 (38.0) | 22 (68.8) | 37 (35.6) | 241 (39.2) |
| Current smoker | 576 (18.8) | 29 (31.9) | 61 (19.6) | 666 (19.3) | 102 (25.7) | 10 (35.7) | 79 (37.1) | 191 (29.9) | 61 (12.7) | 1 (3.1) | 6 (5.8) | 68 (11.1) |
| Missing | 6 (0.2) | - | - | 6 (0.2) | - | - | - | - | 18 (3.8) | 2 (6.2) | 2 (1.9) | 22 (3.6) |
| **Alcohol drinking status, n (%)** |  |  |  |  |  |  |  |  |  |  |  |  |
| Never | 481 (15.8) | 7 (7.7) | 42 (13.5) | 530 (15.3) | 149 (37.5) | 7 (25.0) | 74 (34.7) | 230 (36.1) | 186 (38.8) | 9 (28.1) | 49 (47.1) | 244 (39.7) |
| Low | 1260 (41.2) | 27 (29.7) | 102 (32.8) | 1389 (40.2) | 93 (23.4) | 10 (35.7) | 39 (18.3) | 142 (22.3) | 13 (2.7) | 2 (6.3) | 4 (3.8) | 19 (3.1) |
| Intermediate | 722 (23.6) | 24 (26.4) | 58 (18.6) | 804 (23.3) | 99 (24.9) | 9 (32.1) | 60 (28.2) | 168 (26.3) | 161 (33.6) | 12 (37.5) | 29 (27.9) | 202 (32.8) |
| High | 158 (5.2) | 12 (13.2) | 28 (9.0) | 198 (5.7) | 56 (14.1) | 2 (7.1) | 40 (18.8) | 98 (15.4) | 109 (22.8) | 9 (28.1) | 21 (20.2) | 139 (22.6) |
| Missing | 435 (14.2) | 21 (23.0) | 81 (26.1) | 537 (15.5) | - | - | - | - | 10 (2.1) | - | 1 (1.0) | 11 (1.8) |
| **Fruit and vegetables intake, n (%)** |  |  |  |  |  |  |  |  |  |  |  |  |
| Low | 861 (28.2) | 24 (26.4) | 77 (24.8) | 962 (27.8) | 118 (29.7) | 8 (28.6) | 85 (39.9) | 211 (33.1) | 163 (34.0) | 10 (31.3) | 29 (27.9) | 202 (32.8) |
| Intermediate | 887 (29.0) | 26 (28.6) | 76 (24.4) | 989 (28.6) | 129 (32.5) | 13(46.4) | 71 (33.3) | 213 (33.4) | 168 (35.1) | 5 (15.6) | 34 (32.7) | 207 (33.7) |
| High | 873 (28.6) | 20 (22.0) | 77 (24.8) | 970 (28.1) | 150 (37.8) | 7 (25.0) | 57 (26.8) | 214 (33.5) | 148 (30.9) | 17 (53.1) | 41 (39.4) | 206 (33.5) |
| Missing | 435 (14.2) | 21 (23.1) | 81 (26.0) | 537 (15.5) | - | - | - | - | - | - | - | - |
| **Salt intake, n (%)** |  |  |  |  |  |  |  |  |  |  |  |  |
| Low | 876 (28.7) | 14 (15.4) | 67 (21.5) | 957 (27.7) | 221 (55.7) | 15 (53.6) | 109 (51.2) | 345 (54.1) | 215 (44.9) | 11 (34.4) | 41 (39.4) | 267 (43.4) |
| Intermediate | 913 (29.9) | 15 (16.5) | 63 (20.3) | 991 (28.7) | 56 (14.1) | 7 (25.0) | 52 (24.4) | 115 (18.0) | 207 (43.2) | 15 (46.9) | 52 (50.0) | 274 (44.6) |
| High | 832 (27.2) | 41 (45.1) | 100 (32.2) | 973 (28.1) | 120 (30.2) | 6 (21.4) | 52 (24.4) | 178 (27.9) | 28 (5.8) | 5 (15.6) | 8 (7.7) | 41 (6.7) |
| missing | 435 (14.2) | 21 (23.1) | 81 (26.0) | 537 (15.5) | - | - | - | - | 29 (6.1) | 1 (3.1) | 3 (2.9) | 33 (5.4) |
| **History of diabetes, n (%)** |  |  |  |  |  |  |  |  |  |  |  |  |
| No | 2589 (84.7) | 71 (78.0) | 248 (79.7) | 2908 (84.1) | 368 (92.7) | 28 (100) | 198 (93.0) | 594 (93.1) | 0 (0.0) | 0 (0.0) | 0 (0.0) | 0 (0.0) |
| Yes | 459 (15.0) | 18 (19.8) | 57 (18.3) | 534 (15.4) | 28 (7.1) | 0 (0) | 15 (7.0) | 43 (6.7) | 0 (0.0) | 0 (0.0) | 0 (0.0) | 0 (0.0) |
| Missing | 8 (0.3) | 2 (2.2) | 6 (2.0) | 16 (0.5) | 1 (0.2) | - | - | 1 (0.2) | 379 (100) | 32 (100) | 104 (100) | 615 (100) |
| ***H. pylori* infection status, n (%)** |  |  |  |  |  |  |  |  |  |  |  |  |
| Negative | 223 (7.3) | 8 (8.8) | 8 (2.6) | 239 (6.9) | 0 (0.0) | 0 (0.0) | 0 (0.0) | 0 (0.0) | 0 (0.0) | 0 (0.0) | 0 (0.0) | 0 (0.0) |
| Positive | 1656 (54.2) | 51 (56.0) | 182 (58.5) | 1889 (54.6) | 0 (0.0) | 0 (0.0) | 0 (0.0) | 0 (0.0) | 0 (0.0) | 0 (0.0) | 0 (0.0) | 0 (0.0) |
| Missing | 1177 (38.5) | 32 (35.2) | 121 (38.9) | 1330 (38.5) | 397 (100) | 28 (100) | 213 (100) | 638 (100) | 379 (100) | 32 (100) | 104 (100) | 615 (100) |
| **Family history of GC, n (%)** |  |  |  |  |  |  |  |  |  |  |  |  |
| No | 2859 (93.6) | 78 (85.7) | 255 (82.0) | 3192 (92.3) | 356 (89.7) | 27 (96.4) | 186 (87.3) | 569 (89.2) | 453 (94.6) | 31 (96.9) | 83 (79.8) | 567 (92.2) |
| Yes | 182 (6.0) | 11 (12.1) | 52 (16.7) | 245 (7.1) | 21 (5.3) | 0 (0.0) | 16 (7.5) | 37 (5.8) | 26 (5.4) | 1 (3.1) | 21 (20.2) | 48 (7.8) |
| Missing | 15 (0.4) | 2 (2.2) | 4 (1.3) | 21 (0.6) | 20 (5.0) | 1 (3.6) | 11 (5.2) | 32 (5.0) | - | - | - | - |
| **Histological type, n %** |  |  |  |  |  |  |  |  |  |  |  |  |
| Intestinal | - | 29 (31.9) | 122 (39.2) | 151 (4.4) | - | 20 (71.4) | 128 (60.1) | 148 (23.2) | - | 20 (62.5) | 54 (51.9) | 74 (12.0) |
| Diffuse | - | 11 (12.1) | 85 (27.3) | 96 (2.8) | - | 4 (14.3) | 67 (31.5) | 71 (11.1) | - | 6 (18.8) | 38 (36.5) | 44 (7.2) |
| Mixed/Undifferentiated | - | 3 (3.3) | 16 (5.1) | 19 (0.5) | - | 4 (14.3) | 18 (8.5) | 22 (3.4) | - | 5 (15.6) | 8 (7.7) | 13 (2.1) |
| Missing | - | 48 (52.7) | 88 (28.3) | 136 (3.9) | - | - | - | - | - | 1 (3.1) | 4 (3.9) | 5 (0.8) |

# **Supplementary Figure S1.** Galbraith plot examining heterogeneity between studies in the association between BMI overweight and risk of non-cardia gastric cancer.


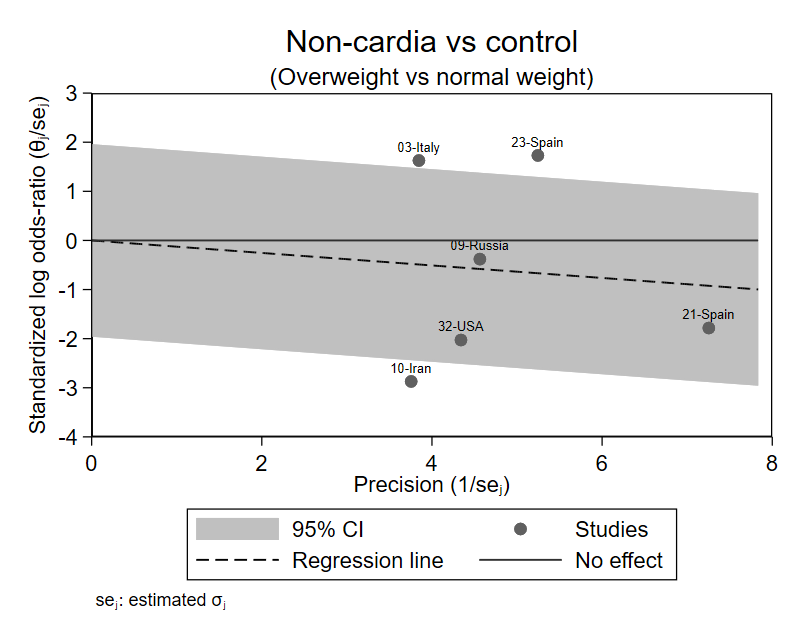


# **Supplementary Figure S2.** Study-specific and adjusted pooled odds ratios (ORs) and corresponding 95% confidence intervals (CIs) for the association between BMI and cardia gastric cancer risk, and separately non-cardia gastric cancer risk using the one-stage approach, and comparison with the two-stage results.

| 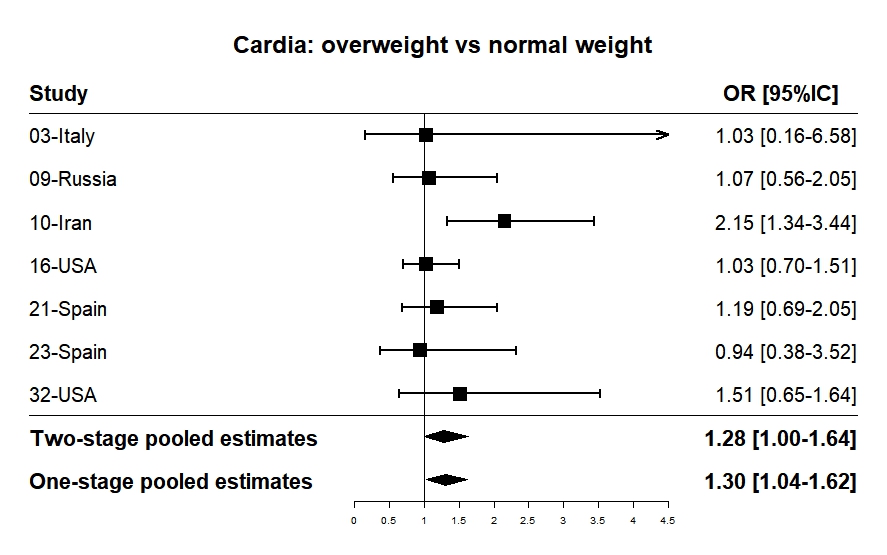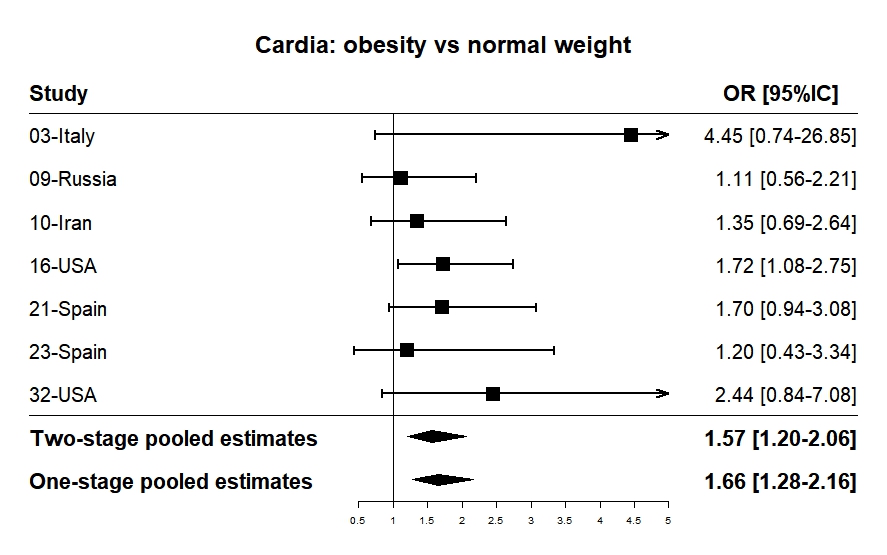 | 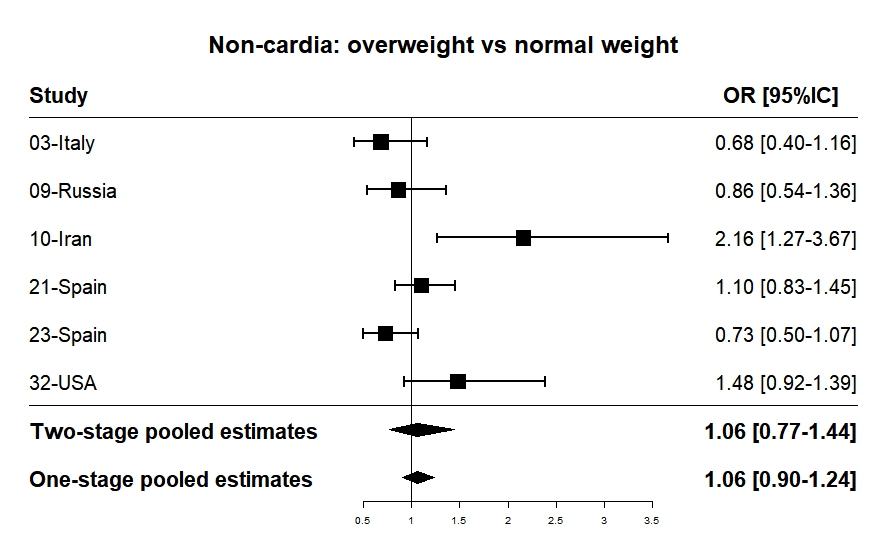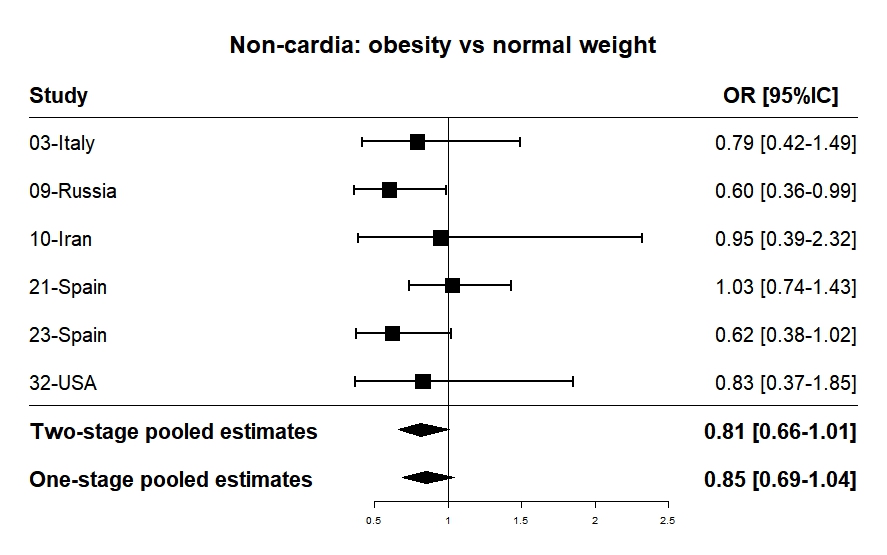 |
| --- | --- |

The number of individuals categorized as underweight (N=94) is insufficient for the meta-analysis.

One stage analysis was justified by ICC values of 0.18 for cardia­ gastric cancer and 0.47 for non-cardia gastric cancer.

# **Supplementary Figure S3.** Odds ratios (ORs) specific to each study and adjusted pooled ORs with corresponding 95% confidence intervals (CIs) for the association between cardia gastric cancer risk and BMI categories (overweight, obesity class I and II/III).


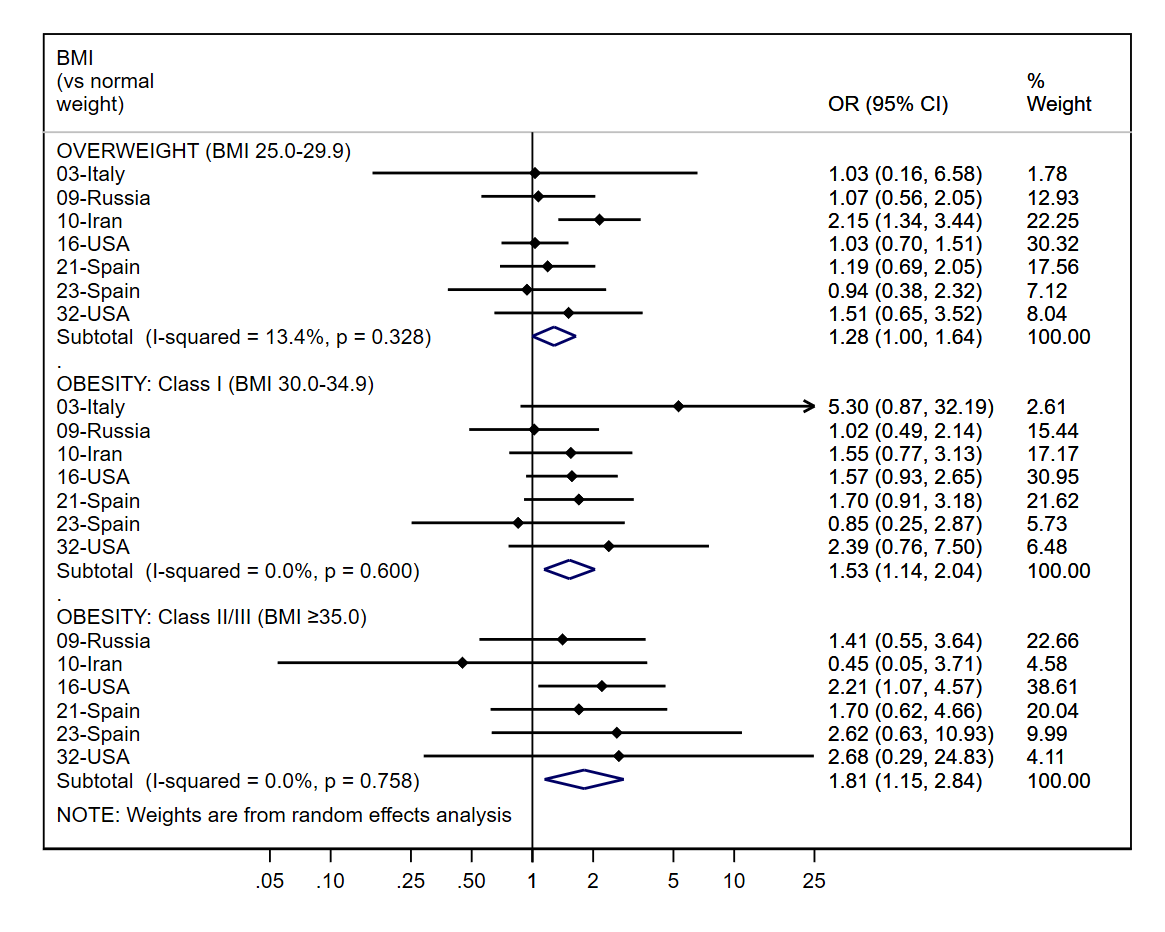


The number of individuals categorized as underweight (N=94) is insufficient for the meta-analysis.

# **Supplementary Figure S4.** Odds ratios (ORs) specific to each study and adjusted pooled ORs with corresponding 95% confidence intervals (CIs) for the association between cardia gastric cancer risk and BMI categories (overweight and obese individuals) using only population-based controls.


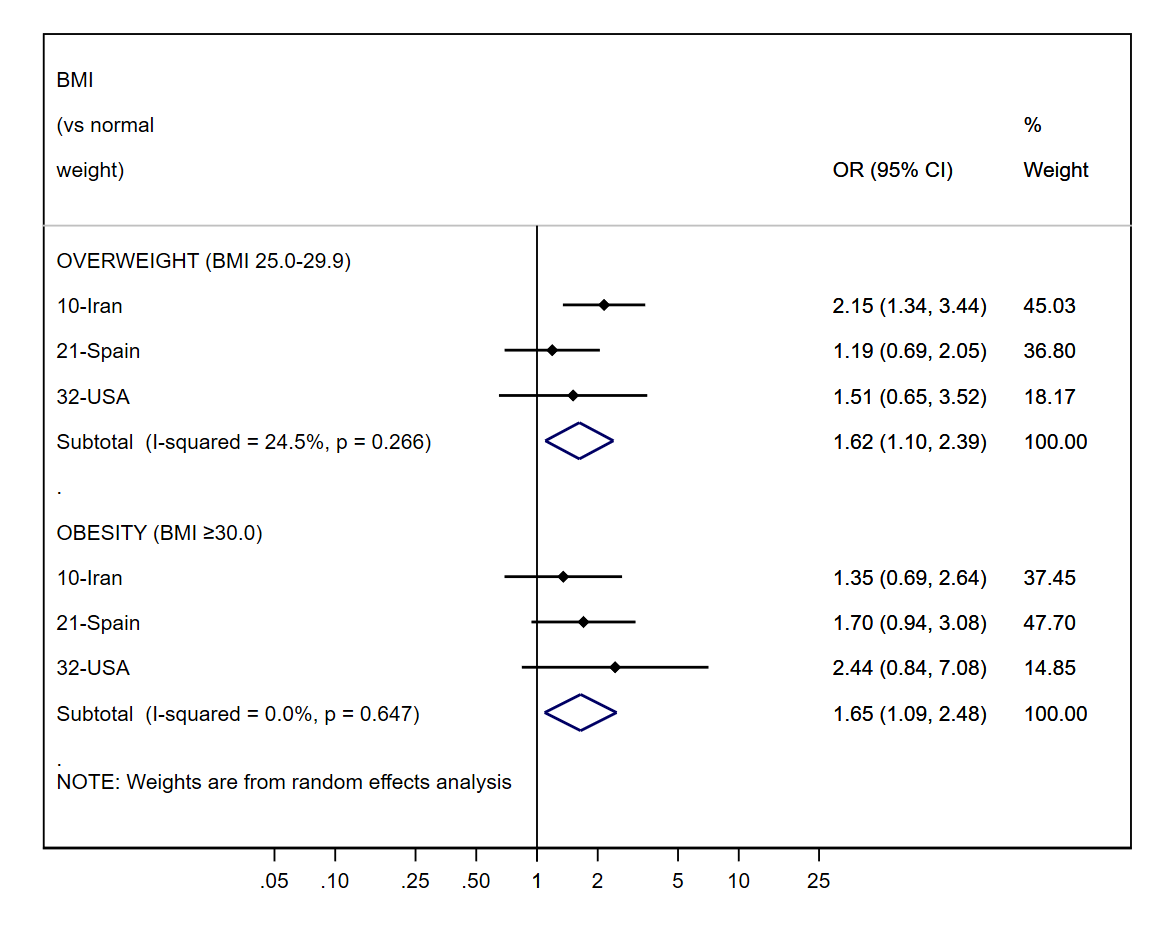


The number of individuals categorized as underweight (N=94) is insufficient for the meta-analysis.
